# Evaluation of Red Cell Membrane Cytoskeletal Disorders Using a Flow Cytometric Method in South Iran

**DOI:** 10.4274/Tjh.2012.0146

**Published:** 2014-03-05

**Authors:** Habib Alah Golafshan, Reza Ranjbaran, Tahereh Kalantari, Leili Moezzi, Mehran Karimi, Abbas Behzad- Behbahani, Farzaneh Aboualizadeh, Sedigheh Sharifzadeh

**Affiliations:** 1 Diagnostic Laboratory, Sciences and Research Technology Center, Shiraz University of Medical Sciences, Shiraz, Iran; 2 School of Para Medical Sciences, Shiraz University of Medical Sciences, Shiraz, Iran; 3 Hematology Research Center, Shiraz University of Medical Sciences, Shiraz, Iran

**Keywords:** RBC, Membrane disorders, Band 3, Flow cytometry

## Abstract

**Objective: **The diagnosis of hereditary red blood cell (RBC) membrane disorders, and in particular hereditary spherocytosis (HS) and Southeast Asian ovalocytosis (SAO), is based on clinical history, RBC morphology, and other conventional tests such as osmotic fragility. However, there are some milder cases of these disorders that are difficult to diagnose. The application of eosin-5’-maleimide (EMA) was evaluated for screening of RBC membrane defects along with some other anemias. We used EMA dye, which binds mostly to band 3 protein and to a lesser extent some other membrane proteins, for screening of some membrane defects such as HS.

**Materials and Methods:** Fresh RBCs from hematologically normal controls and patients with HS, SAO, hereditary elliptocytosis, hereditary spherocytosis with pincered cells, severe iron deficiency, thalassemia minor, and autoimmune hemolytic anemia were stained with EMA dye and analyzed for mean fluorescent intensity (MFI) using a flow cytometer.

**Results:** RBCs from patients with HS and iron deficiency showed a significant reduction in MFI compared to those from normal controls (p<0.0001 and p<0.001, respectively), while macrocytic RBCs showed a significant increase in MFI (p<0.01). A significant correlation was shown between mean corpuscular volume and MFI, with the exceptions of HS and thalassemia minor.

**Conclusion:** Our results showed that the flow cytometric method could be a reliable diagnostic method for screening and confirmation, with higher sensitivity and specificity (95% and 93%, respectively) than conventional routine tests for HS patients prior to further specific membrane protein molecular tests.

## Abstract (tr)

**Amaç:** Kalıtsal eritrosit zarı bozukluklarının ve özellikle kalıtsal sferositoz ve Güneydoğu Asya ovalositozunun tanısı klinik öykü, eritrosit morfolojisi ve ozmotik frajilite gibi konvansiyonel testlere dayanmaktadır. Ancak bu hastalıkların tanı koymada zorlanılan daha hafif formları bulunmaktadır. Eozin-5-malemid’in (EMA) kullanımı diğer anemiler yanında eritrosit zarı bozukluklarının taramasında değerlendirilmiştir. Biz HS gibi bazı zar bozukluklarının değerlendirilmesinde, öncelikle band 3 proteinini ve daha az oranda diğer zar proteinlerini bağlayan, EMA boyasını kullandık.

**Gereç ve Yöntemler:** Hematolojik açıdan normal kontrollerin ve HS, SAO, kalıtsal eliptositoz, kıskaç hücreli kalıtsal sferositoz, ağır demir eksikliği anemisi, talasemi minor ve otoimmun hemolitik anemi hastalarının taze eritrositleri EMA ile boyandı ve akım sitometri kullanılarak ortalama floresan yoğunluğu (MFI) değerlendirildi.

**Bulgular:** HS ve demir eksikliği olan hastaların eritrositleri normal kontrollere kıyasla MFI açısından anlamlı düşüklük gösterirken (sırasıyla p<0,0001 and p<0,001), makrositik eritrositlerde MFI anlamlı olarak yüksekti (p<0,01). HS ve talasemi minor haricinde ortalama eritrosit hacmi ile MFI’nin anlamlı düzeyde ilişkili olduğu gösterildi.

**Sonuç:** Sonuçlarımız HS hastalarının taraması ve doğrulaması için, konvansiyonel rutin testlerden daha yüksek duyarlık (%95) ve özgünlüğe (%93) sahip olan akım sitometrinin zar proteinlerine yönelik ek, özgün moleküler testler öncesinde güvenilir bir tanısal yöntem olabileceğini göstermektedir.

## INTRODUCTION

The normal biconcave discoid shape (donut shape) of the red blood cell (RBC) is maintained by cytoskeletal proteins underneath the membrane, which mainly include the proteins α- and β-spectrin, ankyrin, actin, and bands 4.1 and 4.2. This protein network and its link with the lipid bilayer are essential for RBCs to remain flexible and carry out their physiological functions [1]. A number of disorders including hereditary spherocytosis and elliptocytosis are caused by decreased expression or mutation of cytoskeletal proteins [2]. Genetic defects in various cytoskeletal proteins may cause some disorders such as hereditary spherocytosis (HS), hereditary pyropoikilocytosis (HPP), hereditary elliptocytosis (HE), and stomatocytosis [3].

HS, which is a genetically transmitted (autosomal dominant) form of spherocytosis with sphere-shaped RBCs rather than a biconcave discoid shape, is prone to hemolysis. The genes responsible for HS encode one or more proteins of the cytoskeleton of the RBC membrane, which are ankyrin, spectrin, pallidin (protein 4.2), and band 3 protein (anion exchanger 1) [4]. Inheritance of HS is autosomal dominant in approximately two-thirds of the patients (typical HS), in which ankyrin mutations are the most common cause of HS. The remaining HS cases with nondominant autosomal recessive inheritance are due to defects in either α-spectrin or protein 4.2. Molecular studies have revealed that certain membrane protein defects are tied to specific morphologic results, as in HS with pincered RBCs (HSPR), which is specifically associated with a defect in band 3 [5].

Clinical manifestations of HS are usually marked by evidence of hemolysis with anemia, reticulocytosis, splenomegaly, jaundice, and a positive family history. According to the laboratory findings, evidence of HS includes obvious spherocytes lacking central pallor on peripheral blood smear and increased erythrocyte osmotic fragility. 

HE is inherited in an autosomal dominant pattern and is characterized by the presence of elliptical, elongated erythrocytes on peripheral blood smear [4,6]. HE has a worldwide distribution but is more common in individuals of African and Mediterranean ancestry, presumably because of some resistance of the patients to malaria [6]. HPP is a rare cause of severe hemolytic anemia that has a strong association with HE. Patients with HPP usually present in the early newborn period with severe hemolytic anemia, RBC fragmentation, poikilocytosis, elliptocytosis, and microspherocytosis on peripheral blood smear [7]. Up to one-third of family members of HPP patients have HE. A genetic defect of spectrin has been identified in many cases of HE and HPP [8]. More than 60% of cases of HE are the result of mutations of α-spectrin, while 30% and 5% are the result of mutations of β-spectrin and protein 4.1, respectively [7]. In typical HE patients, the osmotic fragility is normal, but in severe HE and HPP, osmotic fragility is increased [6].

Southeast Asian ovalocytosis (SAO; also known as Melanesian elliptocytosis or stomatocytic elliptocytosis) is an unusual, inherited HE variant found in Malaysia, the Philippines, and other parts of Southeast Asia [9]. Rounded elliptocytes, or ovalocytes, and characteristic stomatocytes with longitudinal slits are found on peripheral blood smear. In these patients a 27-bp genomic deletion leads to deletion of 9 amino acids located at the N-terminal cytoplasmic domain of the band 3 protein [10,11].

RBC membrane protein disorders, particularly HS, are therefore primarily screened by clinical symptoms, family history, and peripheral smear examination accompanied with other laboratory tests such as osmotic fragility and the acidified glycerol lysis test. Nevertheless, these tests have been shown to have lower sensitivity and specificity. Diagnosis of these cell membrane disorders has been shown to be confirmed by sodium dodecyl sulfate-polyacrylamide gel electrophoresis (SDS-PAGE) [12].

Recently, the fluorescence intensity of intact red cells has been measured by a flow cytometric approach using the dye eosin-5’-maleimide (EMA), which binds specifically in higher amounts to lysine-430 on the first extracellular loop of the band 3 protein and in lower amounts to Rh blood group proteins and CD47 of the RBC membrane [13,14].

The aim of this study was evaluation of this flow cytometric method for screening of membrane protein disorders such as HS and SAO from other hematological red cell disorders. An ethics committee approved this study.

## MATERIAL AND METHODS

**Patient and Control Groups**

The patient group included 20 cases of HS, 2 cases of HSPR, 22 cases of HE, 2 cases of HPP, and 2 cases of SAO, along with 36 cases of other RBC disorders including macrocytosis with megaloblastic anemia (14 cases), thalassemia minor (7 cases), severe iron deficiency with mean corpuscular volume (MCV) of lower than 70 fL (13 cases), and autoimmune hemolytic anemia (AIHA) (2 cases). Fifteen healthy subjects with normal hematological parameters and red cell morphology were investigated as a normal control group.

**Laboratory Investigations**

Hematological indices were measured on a Sysmex automated cell counter (Sysmex KX-21, Japan). Red cell morphology was studied on Wright-stained peripheral smears. Diagnostic criteria for HS patients were increased red-cell turnover (reticulocytosis/polychromasia), typical spherocytes, and associated absence of an immune cause (negative direct antiglobulin test or Coombs test). The results of peripheral smears and hematological indices were evaluated by 2 expert scientists. SDS-PAGE analysis of RBC membrane proteins was carried out using a modification of Laemmli’s method [15].

**Flow Cytometric Analysis of Red Cells**

The method described by King et al. [13] was used. Briefly, RBCs were washed twice with phosphate buffered saline (PBS) of pH 7.4. Five microliters of packed RBCs was incubated with 25 µL of EMA (0.5 mg/mL PBS; Fluka, USA) for 1 h at room temperature in the dark. The cell suspension was centrifuged for 1 min in a microcentrifuge and the supernatant containing unbound dye was removed. The labeled RBCs were washed 3 times with 500 µL of PBS-bovine serum albumin (BSA) solution (0.5% BSA in PBS). The RBC pellet was suspended again in 0.5 mL of PBS-BSA solution and a 100-µL aliquot of the cell suspension was added to 1.4 mL of PBS-BSA solution for analysis. Fluorescence intensity, as mean fluorescence intensity (MFI), was determined for 10,000 events in the FL1 channel of a BD FACS caliber flow cytometer (BD FACSCalibur, USA). 

**Statistical Analysis**

The means and standard deviations (SDs) were calculated using SPSS 17.0 for Windows and mean±SD values were compared using a 2-tailed Student t-test. The correlation between MFI and MCV was compared with the Pearson correlation test. A p-value of <0.05 was considered statistically significant.

## RESULTS

**Diagnosis of the Patients**

**Figure 1:**
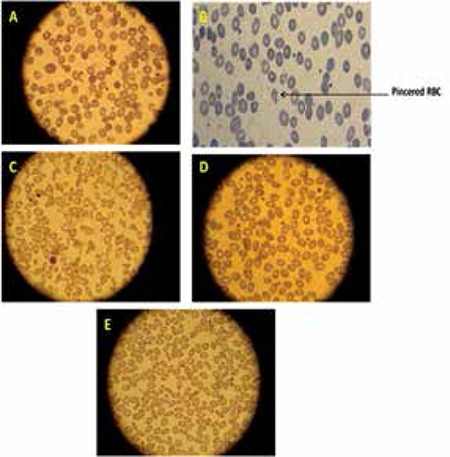
Blood morphology of red cell membrane defects of patients with HS (A), HSPR (B), HPP (C), SAO (D), and HE (E). HS (A) and HSPR (B): The peripheral blood smear of a patient with HS shows spherocytosis, anisocytosis, and polychromasia (increased reticulocytes). Spherocytes are developed due to a loss of RBC membrane, resulting in small, round, dense red cells without central pallor. HPP (C): The red cells of hereditary pyropoikilocytosis demonstrate bizarre forms, anisocytosis, fragments, micropoikilocytosis, microspherocytosis, and budding red blood cells. SAO (D): The red cells of Southeast Asian ovalocytosis are often described as being stomatocytic elliptocytes. The red cells have a slit-like area of central pallor instead of being discocytes. A small proportion of these stomatocytes have 2 transverse slits, giving the appearance of double stomatocytes. HE (E): The peripheral smear of a patient with hereditary elliptocytosis shows elliptical rather than typically biconcave disc-shaped RBCs. Abbreviations: HS: hereditary spherocytosis, HSPR: hereditary spherocytosis with pincered RBC, HPP: hereditary pyropoikilocytosis, SAO: Southeast Asian ovalocytosis, and HE: hereditary elliptocytosis.

According to membrane defect criteria, the results of peripheral smear evaluations are demonstrated in Figure 1. 

**Evaluation of EMA-labeled RBCs of Patients by MFI Level **

**Figure 2:**
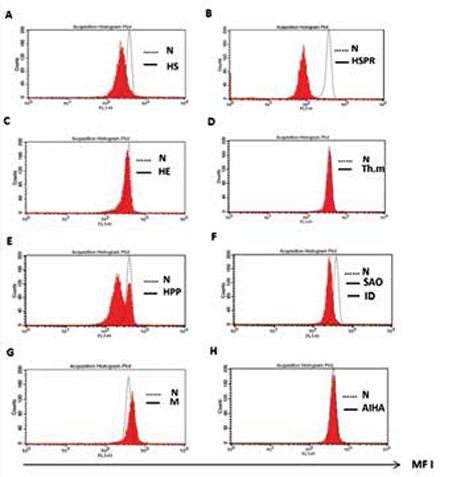
Fluorescence histogram of EMA-labeled RBCs from normal controls and patients with HS (A), HSPR (B), HE (C), Th.m (D), HPP (E), SAO and iron deficiency (F), macrocytosis (G), and AIHA (H). Red blood cells were stained with EMA dye and their intensity was measured as MFI using a flow cytometer. Abbreviations: N: normal control group, Th.m: thalassemia minor, ID: iron deficiency, M: macrocytosis, and AIHA: autoimmune hemolytic anemia.

Fluorescence histograms of EMA-labeled RBCs from normal controls and patients with HS, HSPR, HE, HPP, SAO, macrocytosis, iron deficiency, thalassemia minor, and AIHA are presented in Figure 2. Fluorescence intensity was determined as MFI in the FL1 channel. Labeled red cells from HS, HSPR, HPP, SAO, and iron deficient patient groups showed less fluorescence intensity than those of the normal control group, while labeled RBCs from patients with macrocytosis demonstrated more fluorescence intensity than those of the control group. Only patients with HS, iron deficiency, and macrocytosis showed significant differences compared to the control group (p<0.0001, p<0.001, and p<0.01, respectively).

The data of the normal control group and all patient groups are demonstrated in Table 1. MFI values of RBCs from patients with HS (n=20) and iron deficiency (n=13) were significantly lower than those of the normal group (252±57, p=0.0001 and 280±19, p=0.001, respectively), while the related results of MFI from patients with macrocytosis (n=14) were significantly higher than those from the control group (416±82, p<0.01). MFI values of RBCs from patients with HE (n=22) and thalassemia minor (n=7) were not significantly different from those of the normal controls (335±35, p>0.5 and 344±35, p>0.5, respectively). However, patients with HSPR (n=2), HPP (n=2), and SAO (n=2) showed lower MFIs than the control group, while the MFI values of patients with AIHA (n=2) showed no differences compared to the control group.

**Evaluation of MFI Test**

We checked some statistical tests, such as sensitivity and specificity, positive and negative predictivity, and positive and negative likelihood ratios, to evaluate the MFI test in the HS patient group. The sensitivity and specificity and the positive and negative predictivity of the MFI test were 95%, 93%, 95%, and 93%, respectively. Furthermore, the positive and negative likelihood ratios of the test were 14 and 0.05, respectively. 

**Assessment of MFI/MCV Correlation**

Correlations between MCV and MFI in each group were studied (Table 1). There was a significant correlation between MCV and MFI within each group among the normal control (MCV: 92±4.4, MFI: 337±44, p=0.045), HE (MCV: 84±7.5, MFI: 335±35, p=0.039), macrocytosis (MCV: 113±10.2, MFI: 416±82, p=0.033), and iron deficiency (MCV: 66±5.8, MFI: 280±19, p=0.028) groups. No significant correlation was seen between MCV and MFI in HS (MCV: 88±9.5, MFI: 252±57, p>0.5) or thalassemia minor (MCV: 67±6.6, MFI: 344±35, p>0.5) patients. 

## DISCUSSION

Red cell membrane disorders are generally diagnosed based on clinical history of the disease and peripheral smear examination, along with some routine hematologic tests such as the osmotic fragility test [16]. However, these methods have not shown precise specificity and sensitivity for detection of milder or atypical cases of cytoskeletal disorders. On the other hand, measuring the amount of cytoskeletal proteins in the red cell membrane is difficult, because small variations from normal must be accurately measured. In line with this, some patients with HS have only a 10% to 15% decrease in the affected membrane protein. Therefore, for this purpose, SDS-PAGE of red cell membranes is done in a few specialized laboratories. However, it does not have the required precision and accuracy to measure small reductions in the membrane proteins of mild cases [17].

Our results from SDS-PAGE did not show significant differences between HS patients and the control group. Most of the HS patients had only 10% to 15% or even lower percentages of spherocytes, which may lead to poor diagnosis with SDS-PAGE as the result of small reductions in their affected membrane proteins. This was a motivation to find an easy approach, such as flow cytometry using EMA dye, for diagnosis of cytoskeletal disorders. This method only takes 2 h and has higher sensitivity and specificity than general methods such as osmotic fragility, which is used for screening of HS patients.

Our flow cytometric results from labeled RBCs of HS, HSPR, SAO, HPP, and iron deficiency patients showed lower MFIs as compared to the control group, which was significant only for HS and iron deficiency patients (p<0.0001 and p<0.0001, respectively). According to the MFIs, the labeled RBCs of patients with HE, thalassemia minor, and AIHA showed no significant differences from the control group. On the other hand, patients with macrocytic RBCs showed higher and significantly different MFIs compared to the normal control group (p<0.01). The probable cause of the nonsignificant flow cytometric results of the patients with HSPR (in which their mean MFI was also lower than that of HS patients), SAO, and HPP would be the low number of cases compared to HS. The labeled RBCs of the patients with HPP showed a unique pattern of MFI, with 2 histogram peaks.

The MFIs of HE red cells were similar to normal red cells. Our results are consistent with those of other investigators [13] who found no significant difference in MFIs of red cells between common HE and normal control subjects.

The most common deficiency of cytoskeletal proteins of RBCs in HS patients is ankyrin deficiency, which results in the release of band 3 from low-affinity binding sites on ankyrin [18]. This increased release of band 3 in HS patients causes a reduction of band 3 in their cytoskeletal membrane. The most important cause of genetic defects in HSPR and SAO is a defect in band 3. An abnormal expression of band 3 in iron deficiency anemia has also been shown, which is consistent with our results [19,20]. 

According to the specific binding of EMA dye to band 3, we also measured the correlation between the MCV and MFI of individuals in each group. A significant correlation between them was seen in patients with HE (MCV: 84±7.7, MFI: 335±35, p=0.039), macrocytic RBCs (MCV: 113±10.2, MFI: 416±82, p=0.033), and iron deficiency (MCV: 66±5.8, MFI: 280±19, p=0.028), and in RBCs from healthy controls (MCV: 92±4.4, MFI: 337±44, p=0.045). There was no significant correlation between MCV and MFI in HS patients (MCV: 78.5±9.5, MFI: 250±58, p>0.5) or thalassemia minor patients (MCV: 67±6.6, MFI: 344±35, p>0.5). It seems that there is a direct correlation between MCV and MFI (increased MCV may lead to increased MFI and vice versa), which also results in a direct correlation between MCV and the surface area of RBCs and expression of band 3. Therefore, there is no direct relationship between MCV and MFI in HS and thalassemia minor patients and exceptions to this finding occurred in the HS and thalassemia minor anemia patients. Although spherocytes appear as red blood cells that are smaller than normal on a blood smear (because the diameter of the cells can be assessed on a smear, but noT-cell volume), they have normal volumes and do not have a change in MCV due to cell membrane rigidity. Moreover, in most cases, spherocytes have normal volumes and no change in MCV, while only rare cases show low MCV due to the production of microspherocytes with lower cell volumes. Therefore, in spherocytosis, the defect in RBC membranes and therefore the lowered MFI cannot be affected by the MCV. On the other hand, in spite of the lower MCV in thalassemia minor, which is because of low hemoglobin levels, there is no variation in MFI. Therefore, the normal level of MFI in thalassemia minor patients could be due to absence of a defect in the expression of band 3. 

Some studies have reported variable sensitivity of the osmotic fragility test from 48% to 95%, independent of the cytoskeletal abnormality and of the amount of protein deficiency for diagnosis of HS [21]. However, a sensitivity of 99% was obtained when the osmotic fragility test was done along with the acidified glycerol lysis test on incubated blood [21].

In this study, our emphasis was on patients’ history, and morphological changes in RBCs reported by experts from blood smear examinations can give useful information about RBC membrane defects (Figure 1). All patients suspected of HS with reported spherocytes in their blood smears showed significant decreases in MFI.

Taken together, it seems that the fluorescence dye-based method is a reliable diagnostic assay with higher sensitivity and specificity (95% and 93%, respectively) than conventional routine tests for HS patients.

We propose that this approach will contribute a rapid screening and confirmation test for diagnosis of HS, HSPR, SAO, and HPP patients before performing further specific membrane protein molecular tests.

## ACKNOWLEDGMENT

This study was done at and supported by Shiraz University of Medical Sciences (grant no. 90-5697).

## CONFLICT OF INTEREST STATEMENT

The authors of this paper have no conflicts of interest, including specific financial interests, relationships, and/ or affiliations relevant to the subject matter or materials included.

## Figures and Tables

**Table 1:**
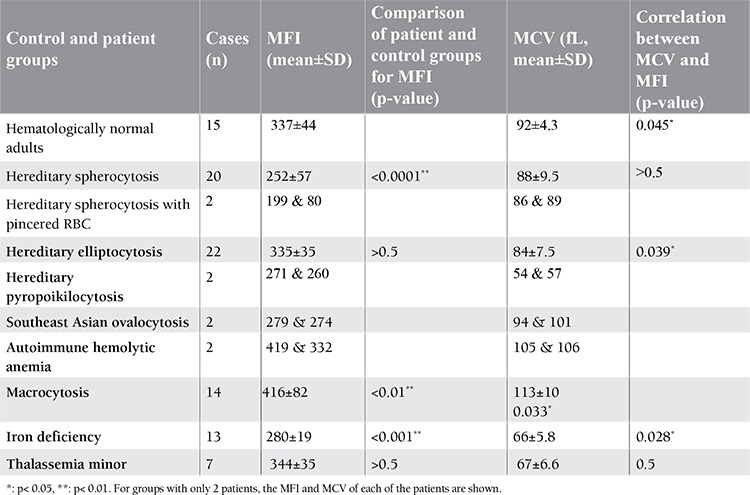
Flow cytometric analysis of EMA-labeled RBCs (MFI) in different hematological disorders and evaluation of correlation between the resultant MFI and MCV
